# Prenatal diagnosis and pregnancy outcomes in fetuses with ventriculomegaly

**DOI:** 10.3389/fmed.2024.1349171

**Published:** 2024-05-09

**Authors:** Fagui Yue, Xiao Yang, Ning Liu, Ruizhi Liu, Hongguo Zhang

**Affiliations:** ^1^Center for Reproductive Medicine and Center for Prenatal Diagnosis, First Hospital, Jilin University, Changchun, China; ^2^Jilin Engineering Research Center for Reproductive Medicine and Genetics, Jilin University, Changchun, China

**Keywords:** fetal ventriculomegaly, chromosomal microarray analysis, prenatal diagnosis, pregnancy outcomes, hotspot CNVs

## Abstract

**Objective:**

Genetic etiology plays a critical role in fetal ventriculomegaly (VM). However, the studies on chromosomal copy number variants (CNVs) in fetal VM are limited. This study aimed to investigate the chromosomal CNVs in fetuses with mild to moderate VM, and explore its genotype-phenotype correlation.

**Methods:**

A total of 242 fetuses with mild to moderate VM detected by prenatal ultrasound were enrolled in our study from October 2018 to October 2022. All cases underwent chromosomal microarray analysis (CMA) and G-banding simultaneously. All VM cases were classified different subgroups according to the maternal age, severity, VM distribution and presence/absence of other ultrasound abnormalities. The pregnancy outcomes and health conditions after birth were followed up. We also performed a pooled analysis regarding likely pathogenic and pathogenic CNVs (LP/P CNVs) for VM.

**Results:**

The detection rate of chromosomal abnormalities by karyotyping was 9.1% (22/242), whereas it was 16.5% (40/242) when CMA was conducted (*P* < 0.05). The total detection rate of chromosomal abnormalities by karyotyping and CMA was 21.1% (51/242). A 12.0% incremental yield of CMA over karyotyping was observed. The detection rate of total genetic variants in fetuses with bilateral VM was significantly higher than in fetuses with unilateral VM (30.0% vs. 16.7%, *P* = 0.017). No significant differences were discovered between isolated VM and non-isolated VM, or between mild and moderate VM, or between advanced maternal age (AMA) and non-AMA (all *P* > 0.05). 28 fetuses with VM were terminated and 214 fetuses were delivered: one presented developmental delay and one presented congenital heart disease. The VM cases with both positive CMA and karyotypic results had a higher rate of termination of pregnancy than those with either a positive CMA or karyotypic result, or both negative testing results (*P* < 0.001).

**Conclusion:**

The combination of CMA and karyotyping should be adopted to improve the positive detection rate of chromosomal abnormalities for VM. The total genetic abnormalities detected using both techniques would affect the final pregnancy outcomes. LP/P CNVs at 16p11.2, 17p13, and 22q11.21 were identified as the top three chromosomal hotspots associated with VM, which would enable genetic counselors to provide more precise genetic counseling for VM pregnancies.

## 1 Introduction

Fetal ventriculomegaly (VM), defined as an atrial diameter of ≥10 mm, is one of the most common central nervous system (CNS) abnormalities observed during pregnancy. As a soft marker in prenatal ultrasound findings, the incidence rate of fetal VM is 0.03–2.20% (1–3). According to the suggestions from Society for Maternal-Fetal Medicine (SMFM), fetal VM could be typically classified into two categories: mild (10–15 mm) or severe (>15 mm) or mild (10–12 mm), moderate (13–15 mm) or severe (>15 mm) (4). In addition, fetal VM could also be divided into unilateral/bilateral VM or isolated/non-isolated VM according to diverse parameters. The probands with VM suffer from a higher risk of psychomotor disorders, autism, epilepsy, schizophrenia, attention deficit disorder (ADHD) and learning disability in childhood (5–7).

The etiology of VM is complicated and extensive, mainly including normal variation, structural CNS abnormalities, congenital fetal infections and genetic conditions (8). Among them, genetic disorders are regarded as a critical causing factor associated with VM, which primarily included chromosomal anomalies (e.g., trisomy 18, trisomy 13, Miller Dieker syndrome) and monogenic syndromes (e.g., X-linked hydrocephalus, Meckel-Gruber syndrome, Joubert Syndrome, Walker-Warburg syndrome) (9). In recent years, chromosomal microarray analysis (CMA), as a first-tier clinical diagnostic test in clinic, has been more and more applied in exploring the genetic etiology in prenatal settings, which have made more chromosomal submicroscopic deletions and duplications detectable. Some chromosomal copy number variants (CNVs) have frequently been reported in VM cases, e.g., 16p11.2 microdeletion, 15q11.2 microdeletion and 1q21.1 microdeletion (10–12). It was estimated that the incidence of chromosomal abnormalities in VM was 9% and the incremental yield of CMA in VM was 11% (1). It has been widely accepted that applying CMA in VM would improve the detection rate of genetic abnormalities in varying degrees.

Given that the studies on genetic etiology of VM using CMA were diverse, the correlation between fetal VM and CNVs was not well described. Fetuses with severe VM usually have poor prognosis, especially when combined with other anomalies. However, fetuses with mild to moderate VM could have a variable prognosis, which would cause increased parental anxiety and make genetic counseling more challenging for the clinicians. Herein, we retrospectively analyzed the clinical data and molecular findings of 242 fetuses with mild to moderate VM, assessed the detection rates of genetic variants in diverse VM subgroups, and investigated the genotype-phenotype correlation of VM, aiming to provide better prenatal counseling and clinical management for such VM cases.

## 2 Materials and methods

### 2.1 Clinical data

This retrospective study was performed from October 2018 to October 2022 and enrolled 242 singleton fetuses with mild to moderate VM diagnosed using prenatal ultrasound. These women were referred to the First Hospital of Jilin University, underwent invasive diagnostic testing via amniocentesis and accepted karyotypic analysis and CMA for genetic analysis. All couples denied consanguineous marriage and also denied any exposure to teratogenic agents, irradiation, or infectious diseases during this pregnancy. All enrolled VM subjects were divided into subgroups according to diverse parameters (maternal age, severity, VM distribution and absence/presence of other ultrasound abnormalities). The VM cases were divided into mild (10–11.9 mm) (*n* = 192) and moderate (12–14.9 mm) (*n* = 50) VM groups according to the recommendation from SMFM. If VM was the only abnormality observed in the pregnancy, it was defined as isolated VM (*n* = 193). If other structural or non-structural anomalies were detected, it was classified as non-isolated VM (*n* = 49). Fetal structural anomalies referred to fetal morphological defects, such as cleft lip and palate, ventricular septal defect, microcephaly, etc. The nonstructural anomalies referred to abnormal amniotic fluid volume, soft markers, etc. 162 fetuses with unilateral VM and 80 with bilateral VM were classified. Clinical data was acquired from electronic medical records in our center, including maternal age, gestational age, genetic testing results, follow up outcomes and so on. The peripheral blood samples were collected after obtaining the written informed consent to confirm the CNVs in the fetuses were inherited or *de novo.* All prospective parents received detailed genetic counseling. The study protocol was approved by the Ethics Committee of the First Hospital of Jilin University (No. 2021-706), and written informed consent was obtained from all the couples.

### 2.2 Cytogenetic analysis

Pregnant women accepted amniocentesis for karyotyping analysis with written informed consent. 30 mL of amniotic fluid cells were collected. 20 mL of amniotic fluid cells were cultured in medium (Catalog #99473, Irvine Scientific, United States) for 1 week. After 8–10 days of culturing, the medium was replaced based on cell morphology. Ten to fifteen primary colonies were examined and colchicine was added to the amniotic fluid cells. Then the cell harvest was carried out. After finishing the drop tablets, routine cytogenetic analysis was performed using G-band metaphases at 400–500 banding resolution, which were prepared from the cultured amniotic fluid cells in accordance with standard protocols in our lab. Twenty metaphases were analyzed for all samples according to the International System for Human Cytogenetic Nomenclature 2016.

### 2.3 Chromosomal microarray analysis (CMA)

**FIGURE 1:**
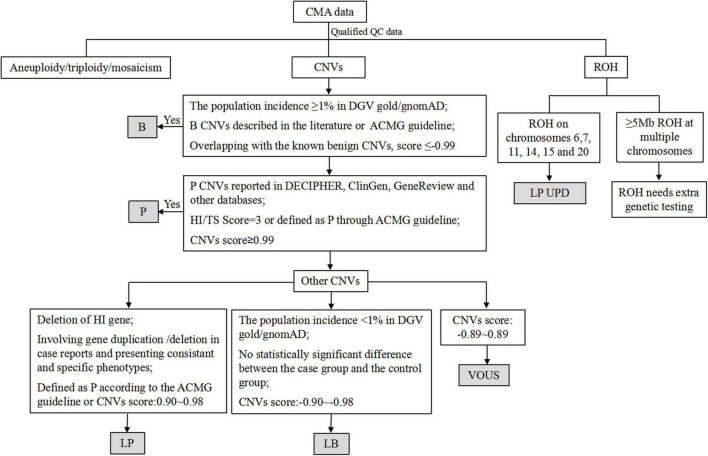
The flow chart of CNVs classification. CNVs, copy number variants; P, pathogenic; LP, likely pathogenic; VOUS, variants of unknown significance; LB, likely benign; B, benign; ROH, region of homozygosity; UPD, uniparental disomy, HI, haploinsufficiency; TS: triplosensitivity.

Following written informed consent from all pregnant women, 10 mL uncultured amniotic fluid cells were collected through amniocentesis. The genomic DNA was extracted from the amniotic fluid cells and parental peripheral blood with QIAamp^®^ DNA Blood Mini Kit (Qiagen, Inc., Hilden, Germany) according to the manufacturer’s protocol. The concentration of gDNA was measured using the Invitrogen Qubit 4.0 (ThermoFisher Scientific). Then the procedures were conducted through CytoScan 750K array (Affymetrix, Santa Clara, CA, USA), in accordance with the manufacturer’s protocol and our previous study (13). The procedure included genomic DNA extraction, digestion and ligation, PCR amplification, PCR product purification, quantification and fragmentation, labeling, array hybridization, washing and scanning. Thresholds for genome-wide screening were set at ≥ 100 kb for gains and losses. The Chromosome Analysis Suite (ChAS) V4.2 software (Affymetrix, California, United States) was used for data analysis. The detected CNVs were comprehensively estimated by comparing them with published literature and the public databases: (1) Database of Genomic Variants (DGV)^1^, (2) Database of Chromosomal Imbalance and Phenotype in Humans using Ensemble Resources (DECIPHER)^2^, (3) Clinical Genome Resource (ClinGen)^3^, (4) ClinVar^4^, (5) PubMed^5^ and (6) Online Mendelian Inheritance in Man (OMIM)^6^. According to the guidelines of American College of Medical Genetics and Genomics (ACMG) (14), all CNVs were classified as pathogenic (P), likely pathogenic (LP), variants of unknown significance (VOUS), likely benign (LB) and benign (B). The flow chart of CNVs classification is shown in Figure 1. Genomic positions refer to the Human Genome assembly Dec.2013 (GRCh38/hg38).

### 2.4 Pooled analysis of frequencies of LP/P CNVs detected in VM cases

In order to better illustrate the distributions and frequencies of likely pathogenic and pathogenic CNVs (LP/P CNVs) in VM cases, we made a literature search on VM cohort articles for integrating these CNVs from inception to 2023. The Chinese language databases (Wanfang Data and China National Knowledge Infrastructure) and English language database PubMed (see text footnote 5) were searched using the combination of the following terms, including fetal ventriculomegaly, cerebral ventriculomegaly, CNVs, chromosomal microarray analysis. Then the frequencies of LP/P CNVs detected in VM cases were summarized.

### 2.5 Follow-up outcomes

The follow up was mainly carried out through telephone interview using customized questionnaire by our center’s follow-up staff after all neonates were delivered. The specific follow up contents included pregnancy outcomes (miscarriages or birth), gestation ages of delivery, sex, birth weight/length, ultrasound findings during the pregnancy period (nervous system, cardiovascular system, craniofacial growth, respiratory system, abdominal abnormalities, urinary system, alimentary system, musculoskeletal system and others) and postnatal health conditions (congenital defects, developmental details and so on).

### 2.6 Statistical analysis

Comparisons among diverse VM subgroups were performed using χ^2^ test or Fisher’s exact analysis using SPSS20.0 software. Statistical significance was identified when *P*-value < 0.05 was considered in the process.

## 3 Results

### 3.1 Study population

**FIGURE 2:**
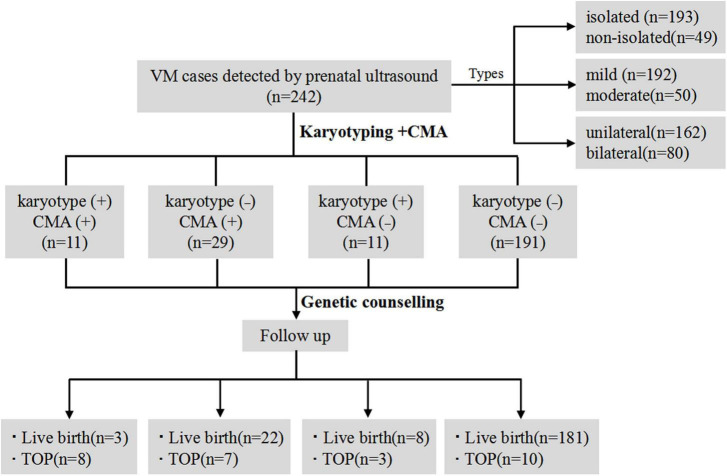
The flow chart of the study. VM, ventriculomegaly; CMA, chromosomal microarray analysis; TOP, termination of pregnancy.

A total of 242 singleton pregnant women who were diagnosed with VM underwent amniocentesis for karyotypic analysis and CMA detection. The mean maternal age was 29.6 ± 4.4 (ranging from 18 to 40) and the mean gestational age was 26.5 ± 3.0 (ranging from 18 to 27) weeks. Karyotypic analysis and CMA were carried out for all VM cases simultaneously. The detection rate of chromosomal abnormalities by karyotyping was 9.1% (22/242), whereas it was 16.5% (40/242) when CMA was conducted (*P* < 0.05). CMA provided a higher positive detection rate of chromosomal abnormalities than karyotyping for all VM cases. The total detection rate of genetic abnormalities by karyotyping and CMA was 21.1% (51/242). Detailed clinical data of VM subgroups and follow-up is shown in Figure 2.

### 3.2 Detection results of chromosomal aberrations in fetal VM

Among all VM cases, 22 fetuses (9.1%, 22/242) were found to have chromosomal abnormalities by karyotyping. All fetuses presenting chromosomal numerical and structural abnormalities were described in Figure 2 and Table 1. Five aneuploidies were identified: trisomy 21 was the most common aneuploidy (*n* = 3), and 47,XXY and 45,X[17]/46,XY[33] were also detected. Meanwhile, seven chromosomal structural anomalies were also discovered, including two deletions involving chromosome 4 and 14, two presenting 46,XN, 2q? and 46,XN,19q?, one balanced translocation and two with derivative chromosomes. In addition, 10 cases with chromosomal polymorphisms were also identified by karyotyping. Except for the three cases (no. 9, 161 and 164), all chromosomal abnormalities detected by karyotyping were confirmed by CMA.

**TABLE 1:** Clinical data of VM fetuses presenting abnormal karyotypes or CMA findings of clinic significance.

Case No.	Age	Gestational age (weeks)	Other ultrasound findings	Classification	Karyotypic analysis	CMA results(GRCh38)	Size (Mb)	Inheritance	Pathogenicity	Pregnancy outcome
**Abnormal karyotypes and abnormal CMA**										
16	36	30+	Polyhydramnios	Non-isolated; mild; unilateral	47,XXY	arr (X) × 2, (Y) × 1	wc	n.a.	P	TOP
46	24	27+	Fetal echogenic bowel	Isolated; moderate; bilateral	46,XN,?der(X)t(X;15) (q27;q12)	Xp22q27(10001-148648479) × 3 15q11.1q12(19974747-26434853) × 1	148.64 6.5	n.a.	LP LP	TOP at 33w
52	39	20+	/	Isolated; mild; bilateral	47,XN,+21	arr(21) × 3	wc	n.a.	P	Live birth at 38w; weight: 2.6 kg length: 46 cm
74	29	24+	/	Isolated; mild; unilateral	46,XN,?del(14)	4p14(35847529-36829026) × 1 14q13.2q21.1(35855957-41801713) × 1	0.981 5.946	mat mat	LB LP	TOP at 30w
91	38	19+	Increased NT; absence of nasal bone	Non-isolated; mild; unilateral	47,XN,+21	arr(21) × 3 16q23.1(77948892-78459054) × 3	wc 0.51	n.a.	P LB	TOP at 26w
171	38	18+	Increased NT	Non-isolated; mild; unilateral	47,XN,+21	arr(21) × 3	wc	n.a.	P	TOP
199	33	29+	/	Isolated; mild; unilateral	46,XN,2q?	1q42.2q44(233385393-248930485) × 3 2q37.2q37.3(236312265-241840106) × 1	15.545 5.528	*de novo* *de novo*	LP P	TOP at 30w
224	29	27+	Fetal echogenic bowel	Non-isolated; moderate; bilateral	47,XX,+del(9)(q13)[62]/45, X[26]/46,X,+9,der(9)t(X;9) (q13;q13)[12]	9p24.3q13(208455-64964986) × 2-3	64.757	n.a.	P	TOP
227	29	20+	/	Isolated; moderate; bilateral	46,XN,del(4)(p15.2)	4p16.2p15.2(4708538-26078927) × 1	21.37	n.a.	P	TOP
**Abnormal karyotypes and normal CMA**										
9	31	28+	/	Isolated; mild; bilateral	45,X[17]/46,XY[33]	arr(1-22) × 2, (X,N) × 1	/	n.a.	/	TOP at 38w
161	32	24+	/	Isolated; mild; unilateral	46,XN,19q?	arr(1-22) × 2, (X,N) × 1	/	mat	/	Live birth at 35w; weight: 4.8 kg length: 51 cm
164	28	26+	/	Isolated; mild; unilateral	46,XN,t(2;10)(q33;p15)	arr(1-22) × 2, (X,N) × 1	/	n.a.	/	Live birth at 39w; weight: 3.1 kg length: 50 cm
**Normal karyotype and abnormal CMA**										
25	26	25+	Persistent left superior vena cava; polydactyly	Non-isolated; mild; unilateral	46,XN	10q11.22q11.23 (45788343-50241236) × 3	4.45	n.a.	LP	TOP
99	31	25+	/	Isolated; mild; bilateral	46,XN	22q11.21(18339130-21446182) × 3	3.1	pat	P	TOP at 29w
123	32	28+	/	Isolated; mild; unilateral	46,XN	15q11.2(22582283-23370622) × 1	0.788	n.a.	P	TOP at 31w

CMA, chromosomal microarray analysis; LB, likely benign; LP, likely pathogenic; n.a., not available; NT, nuchal translucency; pat, paternal; mat, maternal; P, pathogenic; TOP: termination of pregnancy; wc, whole chromosome.

A total of 40 VM cases (16.5%, 40/242) carrying CNVs were detected by CMA, including four aneuploidies, eight LP/P CNVs, 15 VOUS and 13 benign CNVs. The sizes of LP/P CNVs ranged from 0.788 Mb to 148.64 Mb. Among them, two were parental inheritance, one was *de novo* and five were unavailable. The duplicated/deleted size of VOUS ranged from 0.274 Mb to 1.79 Mb. Among them, two were paternal inheritance, three were *de novo*, and eight were unavailable (Table 2).

**TABLE 2:** The clinical data of thirteen fetuses with VM presenting VOUS.

Case	Age	Gestational age (weeks)	Other ultrasound findings	Category	Karyotypic analysis	CMA results (GRCh38)	Size (Mb)	Inheritance	Pathogenicity	Pregnancy outcome
19	28	26+	/	Isolated; mild; unilateral	46,XN	1p36.12p36.11 (23011141-23774413) × 3	0.76	n.a.	VOUS	Live birth at 38 w weight: 2.8 kg; length: 48 cm
85	25	26+	Persistent left superior vena cav; aberrant right subclavian artery	Non-isolated; moderate; bilateral	46,XN	17p13.3(866226-1351655) × 3	0.485	n.a.	VOUS	Live birth at 41w weight: 4.3 kg; length: 52 cm
98	31	25+	/	Isolated; moderate; unilateral	46,XN	3q27.1(184020767-184189131) × 3	0.168	pat	VOUS	Live birth at 38w6d weight: 3.33 kg; length: 50 cm
103	23	25+	FGR	Non-isolated; mild; unilateral	46,XN	16p12.2(21382685-21908606) × 1	0.526	*de novo*	VOUS	TOP
121	30	31+	Fetal intra-abdominal umbilical vein dilatation	Non-isolated; mild; unilateral	46,XN	15q26.3(100858685-101358925) × 3	0.5	n.a.	VOUS	TOP
125	27	30+	/	Isolated; mild; unilateral	46,XN	22q12.3(33754119-34313945) × 3	0.56	pat	VOUS	Live birth at 39w4d weight: 3.6 kg; length: 50 cm
149	26	29+	FGR	Non-isolated; mild; bilateral	46,XN	arr(16) × 2 hmz	LOH	n.a.	VOUS	Live birth at 40w2d weight: 3.65 kg; length: 51 cm
151	25	26+	/	Isolated; moderate; bilateral	46,XN	3q27.1(184005463-184184687) × 3	0.179	n.a.	VOUS	Live birth at 39w6d weight: 4.0 kg; length: 51 cm
157	36	24+	/	Isolated; moderate; bilateral	46,XN	7p21.3p21.2(12754045-14467300) × 3	1.713	n.a.	VOUS	Live birth at 38w2d weight: 3.5 kg; length: 50 cm
158	38	24+	left renal pelvis separation	Non-isolated; mild; unilateral	46,XN	3p25.3p25.2(10092041-11881589) × 3	1.79	n.a.	VOUS	Live birth at 39w weight: 3.4 kg; length: 50 cm
184	38	24+	/	Isolated; mild; unilateral	46,XN	1q21.1(145605589-146044871) × 3	0.439	n.a.	VOUS	Live birth at 39w weight: 3.6 kg; length: 50 cm
218	23	26+	/	Isolated; mild; bilateral	46,XN	9q21.13(72512680-72786655)x1	0.274	*de novo*	VOUS	TOP
226	30	30+	/	Isolated; moderate; bilateral	46,XN	22q11.21(20375099-21110475) × 3	0.406	*de novo*	VOUS	Live birth at 37w weight: 2.6 kg; length: 52 cm
239	33	22+	choroid plexus cysts; ventricular apical thin point	Non-isolated mild; unilateral	46,XN	16p12.2(21394007-21790569) × 1	0.396	pat	VOUS	Live birth at 37w weight: 3.8 kg; length: 50 cm
241	31	28+	/	Isolated; mild; bilateral	46,XN	2q35(216290673-216587706) × 3	0.297	mat	VOUS	Live birth at 38w6d weight: 2.7 kg; length: 50 cm

FGR, fetal growth restriction; LOH, loss of heterozygosity; mat, maternal; n.a., not available; pat, paternal; TOP, termination of pregnancy; VOUS, variants of unknown significance.

Notably, 29 fetuses with normal karyotypes but CNVs were detected using CMA, suggesting a 12.0% additional diagnostic value. Among these cases, 20 duplications and nine deletions were included. Clinically significant LP/P CNVs involving the 10q11.22q11.23, 22q11.21 and 15q11.2 loci were identified in three fetuses (no. 25, 99 and 123), indicating a 1.2% (3/242) incremental yield by CMA. In addition, 14 cases had VOUS CNVs and 12 cases were found to carry B CNVs.

### 3.3 VM subgroup analysis of chromosomal abnormalities

As shown in Table 3, the incidence rates of total genetic variants in fetuses with isolated VM and non-isolated VM were 36/193 (18.7%) and 15/49 (30.0%), respectively. However, there was no significant difference (*P* > 0.05). The detection rates of total genetic variants in fetuses with mild and moderate VM were 38/192 (19.8%) and 13/50 (26.0%), respectively (*P* > 0.05). The detection rates of total genetic variants in fetuses with advanced maternal age (AMA) and non-AMA VM were 8/32 (25.0%) and 43/210 (20.5%), respectively (*P* > 0.05). The detection rate of total genetic variants in fetuses with bilateral VM was significantly higher than in those with unilateral VM (30.0% vs. 16.7%, *P* = 0.017).

**TABLE 3:** Comparisons of detection rates of genetic abnormalities in VM subgroups.

Subgroups	Cases, n	Total genetic variants (n, %)	*P-*value
Isolated VM	193	36, 18.7%	0.067
Non-isolated VM	49	15, 30.6%	
Mild VM	192	38, 19.8%	0.338
Moderate VM	50	13, 26.0%	
Unilateral VM	162	27, 16.7%	0.017
Bilaternal VM	80	24, 30.0%	
Maternal age < 35	210	43, 20.5%	0.559
Maternal age ≥ 35	32	8, 25.0%	

VM, ventriculomegaly.

In the non-isolated VM group, 49 cases underwent karyotyping and CMA detection in parallel. The karyotyping detected three aneuploidies, one complex chromosomal structural anomaly and three chromosomal polymorphisms. CMA identified two LP/P CNVs, three aneuploidies, six VOUS and two B CNVs. The abnormal ultrasound types and chromosomal anomalies detected were shown in Table 4. For the multiple system anomalies, a higher detection rate of genetic variants was observed using CMA (31.0%) compared to karyotyping (16.7%), but no significant differences were observed (*P* > 0.05). In addition, cardiovascular system abnormalities were the most common multi-system anomalies in our non-isolated VM cases.

**TABLE 4:** Chromosomal abnormalities in 49 non-isolated VM.

		Karyotyping			CMA		
US findings	Total	Normal	Abnormal	*P*	Normal	Abnormal	*P*
Single system	7	7	0	0.575	7	0	0.167
≥2 system	42	35	7		29	13	
CVS	16	14	2		12	4	
Urinary system	6	6	0		5	1	
FGR	6	5	1		4	2	
Digestive system	4	3	1		3	1	
Thickened NT	4	2	2		2	2	
Skeletal system	3	3	0		1	2	
Respiratory system	2	2	0		2	0	
CNS	2	2	0		2	0	
Others	5	3	2		2	3	

VM, ventriculomegaly; US, ultrasound; CVS, cardiovascular system; FGR, fetal growth restriction; NT, nuchal translucency; CNS, central nervous system; Others include polyhydramnios, fetal cystic hygroma, nasal bone absence or hypoplasia and dilation of the intrahepatic segment of umbilical vein.

### 3.4 Prenatal and postnatal follow-up assessment

We successfully followed up on all VM cases. 28 fetuses were electively terminated, among which 11 were found with P CNVs, three were found with VOUS CNVs, one was found with LB CNVs, and 13 were found with no CNVs. As shown in Table 4, the rate of TOP was higher in cases with both positive CMA and karyotypic testing results, compared with either positive CMA or karyotypic result or both negative testing results (*P* < 0.001). It is inferred that the total genetic variants detected using CMA and karyotyping would affect the pregnancy decisions and outcomes. Of the 214 delivered newborns, one presented developmental delay, one presented congenital heart disease and the remaining 212 cases had a good prognosis after birth till this writing. Among them, 73.4% (157/214) were females and 25.7% (55/214) were males, and 0.9% (2/214) were not available. It was worth noting that the detection rate of total genetic variants in male and female fetuses with VM was 30/157(19.1%) and 3/55(5.5%), respectively (*P* < 0.05). Since all VM cases were still young, long term follow up should be carried out regularly, especially on neurodevelopmental disorders.

## 4 Discussion

With the application of molecular techniques, an increasing number of studies have focused on exploring the genetic causes of VM. In our study, we investigated the application value and diagnostic utility of CMA detection in mild to moderate VM and compared the detection rates in different VM subgroups. The detection rate of chromosomal abnormalities were 9.1% for karyotyping and 16.5% for CMA, respectively. A 12.0% incremental yield of CMA over karyotyping in the VM cases was observed. In addition, the detection rate of genetic variants in fetuses with bilateral VM was significantly higher than in those with unilateral VM. To our knowledge, this is the first cohort study with detailed follow up on VM in northeast China.

Fetal VM is one of the most common ultrasound findings detected during the pregnancy period, with an estimated incidence of 1% (15). Assessing the VM has been recommended as a routine prenatal check in clinic. Among all the etiologies associated with VM, genetic causes have been proven to play critical roles in these fetal CNS abnormalities. Traditionally, karyotyping has always been the main approach in identifying the chromosomal anomalies. In our cohort study, the overall detection rate of chromosomal abnormality in VM was 9.1% for karyotyping. A previous meta-analysis involving the genetic etiology of VM indicated that the incidence rate of chromosomal abnormalities in fetal VM was ranging from 0 to 25% (1). In recent years, a number of studies have further enriched the incidence rates of chromosomal abnormalities in VM by karyotyping. The prevalence of chromosomal abnormalities in the study of Huang et al was found to be 3.3% (11/334) (16). Xue et al. (10) reported a 6.8% rate of chromosomal abnormalities in 222 VM fetuses (10). Chang et al. (2) reported a 12.1% rate of chromosomal abnormalities in 281 fetuses with VM (2). Among the abnormal karyotypes in fetuses with VM, trisomy 21 was found to be the most common chromosomal aneuploidy (2, 3, 10, 11), which was also observed in our VM cases. As known, chromosomal CNVs smaller than 5 Mb could hardly be identified by karyotyping. With the development of molecular genetic technology, CMA has been gradually applied to detect these chromosomal submicroscopic imbalances due to higher resolution. For our VM cases, the detection rate of chromosomal abnormality using CMA was 16.5%, slightly lower than some studies reporting 17.9–20.6% (2, 17) and higher than other studies reporting 6.2–16.3% (3, 10, 11, 18–20). It has been reported that CMA could yield an additional detection rate as a first-tier diagnostic tool in VM, with an incremental yield ranging from 5 to 26% (1, 8). Some recent studies with larger sample sizes have also described that CMA could provide an additional diagnosis rate of 4.4–10.6% in VM (2, 10, 16). In our study, an improved diagnostic yield of 12% using CMA over karyotyping was discovered, which was slightly higher than those newly reported. In general, more large-cohort prospective studies are still needed to clarify the incidence rates of genetic anomalies in VM.

**FIGURE 3:**
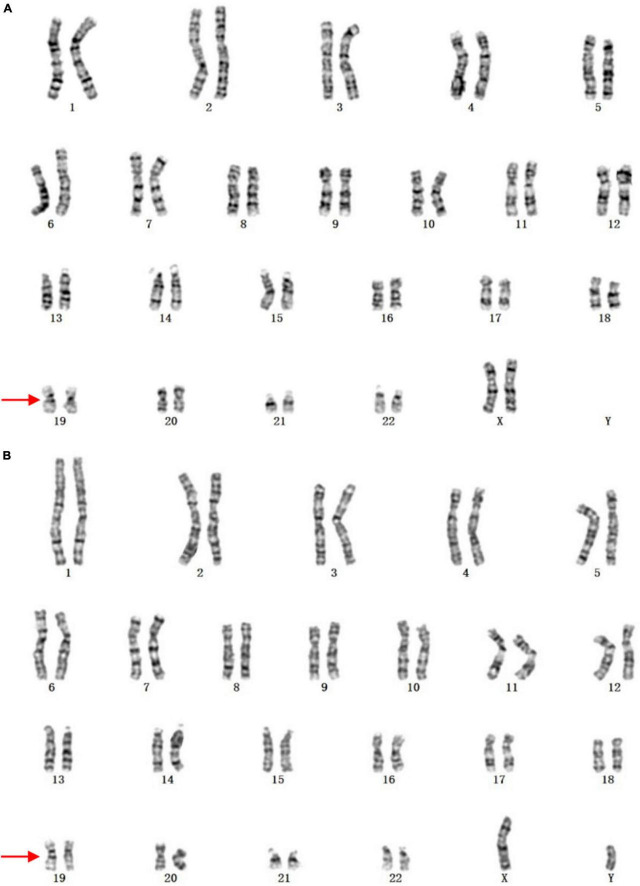
The karyotypes of case 9 and the mother. **(A)** The fetus; **(B)** the mother. The arrows indicate the abnormal chromosomes.

It was worth noting that three cases presenting abnormal karyotypes failed to be identified by CMA. The karyotype of case 9 was 45,X[17]/46,XY[33], with mosaic ratio being 34%. In clinical practice, CMA typically detects uncultured amniocytes directly whereas karyotyping analyzes manual selection of cultured cells. This can lead to fluctuations in chromosome aneuploid mosaicism (21). The karyotype of case 161 was 46,XN,19q?, which was inherited from the mother (Figure 3). It was inferred that this genetic variant in chromosome 19 would not cause bad prognosis for this fetus since no other LP/P CNVs were detected. In addition, CMA can not detect the balanced chromosomal translocations in case 164. Despite the fact that CMA was highly recommended for prenatal diagnosis of fetal VM (3, 11), we currently recommend that it should be used in combination with karyotyping to provide more comprehensive genetic counseling.

Currently, the incidence rates of genetic variants in diverse fetal VM remain controversial in published literature. Hence, we divided our VM cases into diverse subgroups and compared the overall detection rates of chromosomal abnormalities among them. Consistent with the reported data from Wang et al. (11) and Tao et al. (22), the incidence of chromosomal abnormalities in fetuses with moderate VM (26.0%) is slightly higher than in those with mild VM (19.8%), but no significant differences were observed. The bilateral VM group showed significantly higher detection rate of genetic variants than the unilateral VM group in our study, and similar findings were also described in other studies (2, 10, 11). However, the study by Toren et al. indicated that the rate of genetic aberrations was not associated with the degree of dilatation or laterality (23). Multiple studies have shown that the incidence of chromosomal abnormalities was higher in non-isolated VM than in isolated VM (2, 10, 11, 16, 22, 24). However, no significant differences were found in our study and other three studies (10, 22). Notably, the study by Gezer et al. found higher rates of chromosomal abnormalities in isolated VMs (8.6%) compared to non-isolated VMs (3.8%) (25). The detection rate of genetic abnormalities in AMA groups was higher than that in non-AMA groups, but no significant differences were observed. Hence, more studies are needed to analyze the potential influence of maternal age in VM cases. In addition, it was assumed that the male fetuses were prone to be VM. The ratio of males to females in our postnatal VM cases was 2.85:1. The CNVs frequency using CMA in males is higher than that in females (*P* = 0.016) while no statistical significance were observed by karyotyping analysis (*P* > 0.05), which was similar to the results from Xue et al. (10). In the non-isolated VM group, the detection rate in multisystem was higher than that in single system anomalies (Table 5), which was also discovered in other reports (2, 22). The cardiovascular system abnormalities were the most common multi-system anomalies in our study, which was consistent with the findings from Tao et al. (22). Generally speaking, the sample size, sample selection bias, and array platforms could be responsible for detecting discrepancies among the studies, so more studies should be conducted using large-scale sample sets.

**TABLE 5:** The rates of TOP for VM cases with diverse testing results.

Pregnancy outcome	Total	CMA(+) karyotype(+)	CMA(+) karyotype(-)	CMA(-) karyotype(+)	CMA(-) karyotype(-)	χ^2^	*P-*value
TOP	193	72.7% (8/11)	24.1% (7/29)	27.3% (3/11)	5.2% (10/191)	38.448	<0.001

CMA, chromosomal microarray analysis; TOP, termination of pregnancy; VM, ventriculomegaly.

**FIGURE 4:**
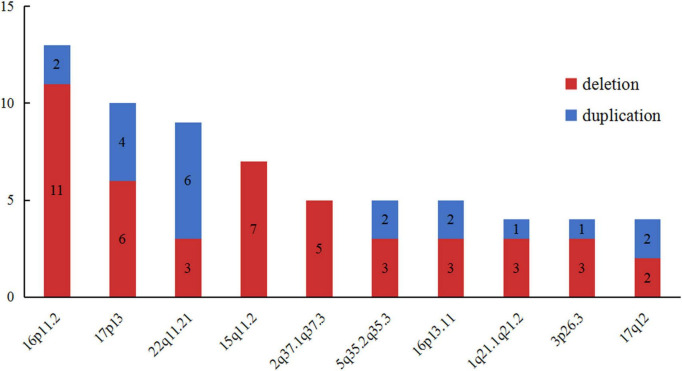
The top ten high-frequency CNVs detected in published VM corhort studies. VM, ventriculomegaly; CNVs, copy number variants.

In order to further delineate the correlation between CNVs and VM, we made a literature review on VM cases carrying LP/P CNVs excluding common aneuploidies (2, 3, 7, 10–12, 16–20, 24, 26–28). A total of 231 LP/P CNVs in published VM cohort studies were collected. Meanwhile, we made a pooled analysis for these recurrent loci, aiming to specify the LP/P CNVs associated with VM. As shown in Figure 4, the top ten high-frequency CNVs in VM cases are as follows: 16p11.2 (*n* = 15), 17p13 (*n* = 10), 22q11.21 (*n* = 9), 15q11.2 (*n* = 7), 2q37(*n* = 5), 5q35.2q35.3 (*n* = 5), 16p13.11 (*n* = 5), 1q21.1q21.2 (*n* = 4), 3p26.3 (*n* = 4), and 17q12 (*n* = 4). Further analysis showed that 16p11.2 deletion, 15q11.2 deletion and 22q11.21 duplication were the top three chromosomal LP/P CNVs, which might be the hotspot CNVs associated with VM.

The genotype-phenotype correlation involving VM is not well delineated. In our study, three VM cases (1.2%, 3/242) with normal karyotypes and LP/P CNVs were confirmed, including the 10q11.22q11.23, 22q11.21 and 15q11.2 regions. Our case 25 presented VM, persistent left superior vena cava and polydactyly. A 4.45 Mb duplication of 10q11.22q11.23 was detected using CMA. Tritto et al. reported a 6-year-old boy with 10q11.22q11.23 duplication presenting autism spectrum disorder, intellectual disability, developmental delay, hypotonia, gross-motor skills deficit, overgrowth and mild dysmorphic features. They proposed that 10q11.22q11.23 duplication was correlated with autism (29). According to the DECIPHER database and published literature, two morbid genes (*WDFY4* and *OGDHL*) in this locus were closely associated with neurodevelopmental diseases. *WDFY4* gene is regarded as a strong candidate autism spectrum disorder (ASD) gene (30). The homozygous or compound heterozygous mutations in the *OGDHL* gene would cause Yoon-Bellen neurodevelopmental syndrome, which is characterized by developmental delay with varying degrees of impaired intellectual development (31). A paternally inherited 22q11.21 duplication was detected in our case 99 presenting isolated VM. The patients carrying 22q11.2 duplication are at increased risk of intellectual disability/learning disability, delayed psychomotor development, growth retardation, muscular hypotonia, ADHD and dysmorphic features (32, 33). *TBX1* gene was recognized as a critical gene in this region. Overexpression of *TBX1* gene might be responsible for the variable phenotypes of dup22q11.2 disorders. Further research is still necessary. Case 123 exhibiting isolated VM was found to carry a 0.788 Mb deletion in the region of 15q11.2 BP1-BP2. The carriers could present developmental and language delay, neurobehavioral disturbances and psychiatric problems in clinic (34). This region covered the *TUBGCP5*, *CFYIP1*, *NIPA1* and *NIPA2* genes, which were critical genes causing behavioral and academic differences in the Prader-Willi/Angelman syndrome. The *NIPA1* gene is highly expressed in the brain and its mutations would cause autosomal dominant hereditary spastic paraplegia and postural disturbance. The mutations of *NIPA2* gene would lead to childhood absence epilepsy. The *TUBGCP5* gene is associated with ADHD and obsessive compulsive disorder (OCD). The product of *CYFIP1* would interact with Fragile X Messenger Ribonucleoprotein, which would cause intellectual disability (35). In general, more studies should be conducted to establish a clear genotype-phenotype correlation for VM.

At present, genetic counseling on prenatally detected VOUS is challenging, which would cause different levels of parental anxiety and affect the final pregnancy decisions. A total of 15 VOUS were detected in our study. 12 out of 15 cases chose to continue their pregnancies and gave birth to children at term. Among them, 10 duplications, one 16p12.2 deletion with paternal inheritance and one loss of heterozygosity (LOH) on chromosome 16 were identified. These cases were in a healthy state after birth and no evident congenital anomalies were observed till this writing. Among the 3/15 abortuses, two carried *de novo* chromosomal deletions involving the regions of 9q21.13 and 16p12.2. It seemed that when VOUS occurs in chromosomal duplication, the couples were more likely to continue their pregnancies, but further research is needed to confirm this. In recent years, prenatal exome sequencing (ES) has been more and more applied in prenatal diagnosis. It was reported that prenatal ES would provide an incremental yield of 31% for fetuses with structural abnormalities when CMA/karyotype analysis is non-diagnostic (36). For severe bilateral VM cases, there was an apparent incremental diagnostic yield of prenatal ES when CMA results were negative (37). Prenatal ES may also play a role in detecting mild to moderate VM, but further research is needed.

There are some limitations in our study. First, the enrolled sample size is relatively small. To delineate a detailed correlation between VM subgroups and CNVs detection rates, the sample size should be enlarged through multi-center collaborations. Second, since all postnatal VM cases are still young at the time of writing, long-term follow-up should be conducted regularly to detect any emerging abnormal clinical phenotypes, especially neurodevelopmental disorders. In addition, the detected anomalies are not validated by a second technique, such as fluorescence *in situ* hybridization (FISH). Further validation is necessary for these genetic variants. Since monogenic syndromes are also associated with VM, further investigation using prenatal ES might be beneficial for revealing the underlying causes, especially when CMA and karyotyping fail to identify pathogenic genetic variants.

In conclusion, our study provides a 12.0% incremental yield of CMA over karyotyping in mild to moderate VM, thereby enhancing the application value of CMA in VM. CNVs detection combined with karyotyping is still an effective approach for exploring the genetic etiology of VM. LP/P CNVs at 16p11.2, 17p13, and 22q11.21 were identified as the top three chromosomal hotspots associated with VM, which would enable genetic counselors to provide more precise genetic counseling for VM pregnancies. However, more evidence should be accumulated to establish a more detailed genotype-phenotype correlation in VM. Due to the uncertainty of prognosis for mild to moderate VM, our study would help clinicians better understand the clinical characteristics and provide a more accurate estimation in the prenatal counseling process for such cases. For VM cases after birth, their postnatal growth and neurodevelopmental problems should be followed up regularly to detect any emerging abnormal phenotypes.

## Data availability statement

The original contributions presented in this study are included in this article/supplementary materials, further inquiries can be directed to the corresponding author.

## Ethics statement

The studies involving humans were approved by the Ethics Committee of the First Hospital of Jilin University. The studies were conducted in accordance with the local legislation and institutional requirements. The participants provided their written informed consent to participate in this study.

## Author contributions

FY: Conceptualization, Investigation, Writing – original draft. XY: Data curation, Formal analysis, Methodology, Writing – original draft. NL: Data curation, Formal analysis, Methodology, Writing – original draft. RL: Funding acquisition, Project administration, Writing – review and editing. HZ: Conceptualization, Supervision, Validation, Visualization, Writing – review and editing.

## Footnotes

1
http://dgv.tcag.ca/dgv/app/home

2
http://decipher.sanger.ac.uk/

3
http://www.clinicalgenome.org/

4
https://www.ncbi.nlm.nih.gov/clinvar/

5
http://pubmed.ncbi.nlm.nih.gov/

6
http://www.ncbi.nlm.nih.gov/omim
